# The numbers behind success: key metrics for advancing in male EURO 2024

**DOI:** 10.3389/fspor.2025.1622887

**Published:** 2025-08-07

**Authors:** Adrián Martín-Castellanos, Marcos Raphael Pereira-Monteiro, Francisco Hermosilla-Perona, Manuel Barba-Ruíz, Diego Muriarte Solana, Enrique Alonso-Pérez-Chao

**Affiliations:** ^1^ Facultad de Ciencias Biomédicas y de la Salud, Universidad Alfonso X el Sabio (UAX), Madrid, Spain; ^2^ Graduate Program in Physiological Sciences, Federal University of Sergipe, Sergipe, Brazil; ^3^ Facultad de Ciencias de la Vida y la Naturaleza, Universidad Nebrija, Madrid, Spain; ^4^ Departamento de Deportes, Facultad de Ciencias de la Actividad Física y el Deporte, Universidad Politécnica de Madrid, Madrid, Spain; ^5^ Department of Real Madrid Graduate School, Faculty of Medicine, Health and Sports, Universidad Europea de Madrid, Madrid, Spain

**Keywords:** football, KPI, performance analysis, soccer, UEFA

## Abstract

This study aimed to identify the key performance indicators (KPIs) that distinguish football teams advancing through the group and knockout stages in the UEFA EURO 2024 tournament. A total of 51 matches were analyzed, including 66 variables sourced from official UEFA statistics. Principal Component Analysis (PCA) was applied to reduce the dataset, retaining variables with eigenvalues greater than 1 and factor loadings of at least 0.6, resulting in a final selection of 37 KPIs. These variables were grouped into five categories: distribution, attacking, defending, goalkeeping, and disciplinary. Generalized linear models were used to compare team performance based on progression status in each phase. In the group stage, advancing teams showed significantly better performance in variables such as Goals Conceded and Passes Completed Backward. In the knockout stage, new decisive KPIs emerged, including Pass Accuracy, Goals Scored Inside the Penalty Area, Assists, Solo Runs Into Key Play Area, and Tackles. Effect sizes for variables like Ball Possession, Attacks, and Goalkeeping metrics increased, highlighting their growing influence. Overall, effective distribution, minimal goals conceded, and offensive effectiveness were critical for progression. While discipline was relevant during the group stage, its importance diminished in the knockout rounds. These results provide practical implications for coaches and analysts, emphasizing the importance of strategic flexibility and data-driven preparation throughout different stages of tournament play.

## 1 Introduction

Football is a complex sport in which teams can achieve victory without registering a single shot on target, for example, by benefitting from an own goal by the opponent (1). This unpredictability highlights the multifaceted nature of the game and the importance of understanding the factors that influence performance beyond the obvious metrics (2). The male European Championship (EURO), organized by Union of European Football Associations (UEFA), held its first edition in 1960 and has had 17 editions (it takes place every four years), with the latest one being in Germany in 2024. Since then, the format has evolved into the current one, where since 2016 there have been 24 teams in the final phase, with six groups of 4 teams. The top two teams from each group, along with the four best third-placed teams, advance to a knockout round starting with the Round of 16. This tournament brings together the strongest national teams in Europe, making it a key reference point within elite football. In this regard, each match in the EURO is crucial, as the tournament's knockout format means that even a single result can determine a team's continuation or elimination. This structure adds pressure and strategic complexity, making it an ideal context for studying the key performance indicators (KPI) that influence high-level football performance.

Analysing these KPI allows teams to better prepare for the demands of high-level competition. Every sport has its own set of KPIs that provide valuable insights into the critical elements driving success. KPIs are used to assess performance and identify areas for improvement, helping coaches, analysts, and players focus on the most impactful aspects of the game (3). Historically, different approaches have been made to identify the most important KPI and whether they vary depending on the competition, outcome or match venue (4–8), along with the original areas to which they belong: technical, tactical, or physical (9–12). Beyond goals (13), even small factors such as a higher number of corners in winning teams (14), greater passing and offensive efficiency (11, 15, 16), or the transition play after losing the ball (11) can make the difference between advancing or being eliminated.

However, the analysis of these successful variables has often been used to understand differences between winners and runners-up (6) or applied in longer tournaments, such as domestic leagues (3, 8, 17). In the context of international competitions, comparisons between confederations can be observed (18, 19) as well as groupings based on rankings (7, 20) or the number of matches played (5). Nevertheless, there is still a lack of comprehensive studies that examine international tournaments integrating both the group stage and the knockout phase. The most comparable analyses of group and knockout phases, such as that conducted by Dufour et al. (21) or Stafylidis et al. (22), lacked the inclusion of data starting from the elimination rounds of the competition. As a result, determining whether these KPIs could vary throughout such tournaments remains a significant challenge.

Although the group and knockout stages may appear similar at first glance, the context in which teams compete differs considerably, despite the shared ultimate objective: winning. In the group stage, the aim is to accumulate as many points as possible across multiple matches, which, if achieved early, may allow for squad rotation in the final fixture. In contrast, the knockout stage demands a definitive outcome, often leading to more aggressive tactical approaches and increased psychological pressure on players. These contextual differences may influence which performance indicators are most relevant at each stage of the competition. Therefore, this study aimed to identify the key performance indicators that differentiate teams that advanced from those that were eliminated during both the group stage and the knockout rounds of the EURO 2024.

## 2 Method

This study followed an observational and comparative design, based on the analysis of official performance data from all matches played during the group and knockout stage of UEFA EURO 2024. To collect the match data required for the analysis, an automated web scraping procedure was implemented. An HTTP GET request was sent to retrieve the HTML content of each match webpage, allowing access to the tags and elements containing the variables of interest. For the comprehensive analysis of all matches played during the UEFA EURO tournament, a list was compiled with the URLs corresponding to each match. The data extraction function, previously defined, was then applied iteratively to this list in order to systematically retrieve and structure the relevant information.

The following Python libraries were used for this procedure: ‘requests’ for managing HTTP requests, “beautifulsoup” for parsing and navigating the HTML structure, “selenium” for dynamic content handling, and “pandas” for organizing the extracted data into structured dataframes suitable for analysis. The data was exported to a Microsoft Excel spreadsheet (version 16.0; Microsoft Corporation, Redmond, WA) for subsequent analysis.

### 2.1 Research group

A total of 51 matches were analyzed during the final phase of EURO 2024, held between June 14 and July 14, featuring 24 teams. We examined variables from both competing teams, creating a comprehensive database of 102 records. Regarding the matches in the tournament, 36 group-stage matches, and 15 knockout-stage matches were played. Of the group-stage games, 23 were decided within regular time or extra time, while in the knockout phase, 12 matches were resolved without requiring a penalty shootout. Only 3 matches in the knockout stage were decided by penalties shootouts. It is important to note that all matches included in the analysis were required to be part of the final phase of the tournament, thereby excluding any pre-classification matches. Furthermore, all matches from the final tournament phase were considered, regardless of factors such as total playing time, red cards, or other in-game incidents.

### 2.2 Variables

The data were obtained from the official website of the UEFA, available from https://www.uefa.com/euro2024/fixtures-results/#/d/2024-07-10 (23). A total of 66 variables were extracted, and grouped into five main categories: attacking, distribution, defending, goalkeeping, and disciplinary. Attacking variables comprised goals (scored inside and outside the penalty area), total attempts, attempts on target and off target, blocked shots, attempts hitting the woodwork (crossbar or post), assists, penalties (awarded, scored, and missed), total attacks, clear chances, corners taken, offsides, dribbles, and runs into key offensive zones such as the attacking third, the key play area, and the penalty area. Distribution variables included ball possession percentage, passing accuracy, passes attempted and completed (including short, medium, long, and backward passes), directional passes (to the left and right), free-kicks taken, and passes into the attacking third, key play area, and penalty area, as well as crossing accuracy, crosses completed and attempted, and total instances of possession. Defending variables encompassed balls recovered, blocks, penalties conceded, total tackles (won and lost), and clearances (completed and attempted). Goalkeeping performance was assessed through goals conceded, own goals, clean sheets, saves (including those from direct free-kicks, indirect free-kicks, and penalties), claims (high and low), and punches made. Finally, disciplinary variables included yellow and red cards, total fouls committed, and fouls committed in both the defensive third and own half. This classification is based on the variable taxonomy and performance dictionary promoted and used by UEFA for official match analysis.

To reduce the dimensionality of the dataset and identify potentially relevant variables, a principal component analysis was conducted to extract the main components explaining the overall variance, using orthogonal Varimax rotation to improve interpretability. Factors with eigenvalues below 1 and variables with loadings less than 0.6 were excluded (24). The Kaiser–Meyer–Olkin test was used to measure sampling adequacy, resulting in a rate of appropriateness (.791). Bartlett's test of sphericity was performed (*χ^2^* = 5116.36; *df* = 666; *p* < .001), and the percentage of total variance explained was 85.49%.

A total of 37 variables were finally included, organised into different categories: Distribution (Ball Possession, Free Kick, Delivery Into Attacking Third, Delivery Into Key Play Area, Cross Attempted, Cross Accuracy, Cross Completed, Passes Attempted, Passes Completed, Passes Accuracy, Passes Short Completed, Passes Medium Completed, Passes Completed Backward, Passes Completed to Left, and Passes Completed to Right), Attacking (number of Attacks, Corners, Goals, Goals Scored Inside Penalty Area, Attempts Off Target, Attempts Off Target Outside Penalty Area, Attempts On Target Outside Penalty Area, Assists and Runs Solo Into Key Play Area) Defending (Tackles, Tackles Won, Clearance Attempted and Completed), Goalkeeping (Goals Conceded, Clean Sheets, Claims, Claims High, Claims Low and Punches) and Disciplinary (Fouls Committed, Fouls Committed in Defensive Third and Fouls Committed in Own Half).

To conduct a comprehensive comparison of the group stage, teams were classified based on their advancement to the knockout stage. This analysis encompassed all matches played by each team within these respective categories. In the knockout stage, considering the elimination format—wherein a team is eliminated upon losing—the focus shifted to identifying match winners and losers. This approach allowed for a clear understanding of the distinctions between teams that successfully advanced to the next round and those that were eliminated.

### 2.3 Statistical analysis

Descriptive data were presented as means and standard deviations. To analyze differences across groups in both the group stage and knockout phase scenarios, we used a Generalized Linear Model (GLM) with a Gaussian distribution and identity link function. In the group stage scenario, the model included progression beyond the group stage (qualified vs. non-qualified teams) as the independent factor. In the knockout phase scenario, the model used match outcome (winning vs. eliminated teams) as the independent factor. This modeling approach was selected over traditional ANOVA to allow for greater flexibility in handling potential violations of assumptions such as normality and homoscedasticity. We also tested alternative GLM specifications (e.g., using a Gamma distribution), but based on Akaike Information Criterion (AIC) values and the presence of zero values in some variables, the Gaussian distribution was retained. Partial *η*2 was calculated (25) for the interactions using the results by GLM. Effect sizes were interpreted based on the following thresholds (26): small effects (*η*² < 0.01), medium effects (*η*² between 0.02 and 0.14), and large effects (*η*² > 0.14). For pairwise comparisons, the effect size was estimated using Cohen's d (26), where values of 0.2, 0.5, and 0.8 are considered small, medium, and large effects, respectively. All calculations were performed using Jamovi software version 2.6, while graphs were generated using GraphPad Prism software version 8.0.

## 3 Results

**Figure 1:**
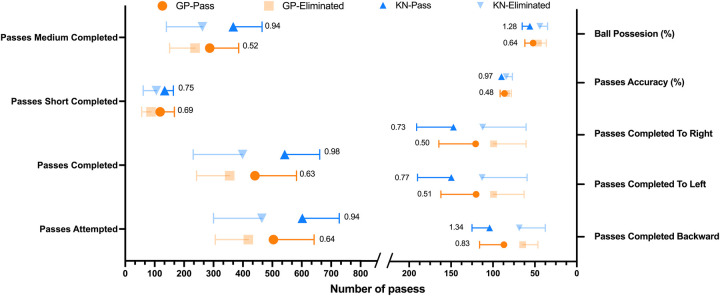
Significant variables related to distribution (passes) included in the generalized linear model. Values inside de graph represent the effect size (*η*^2^p); GP, group phase; KN, knockout phase.

**Figure 2:**
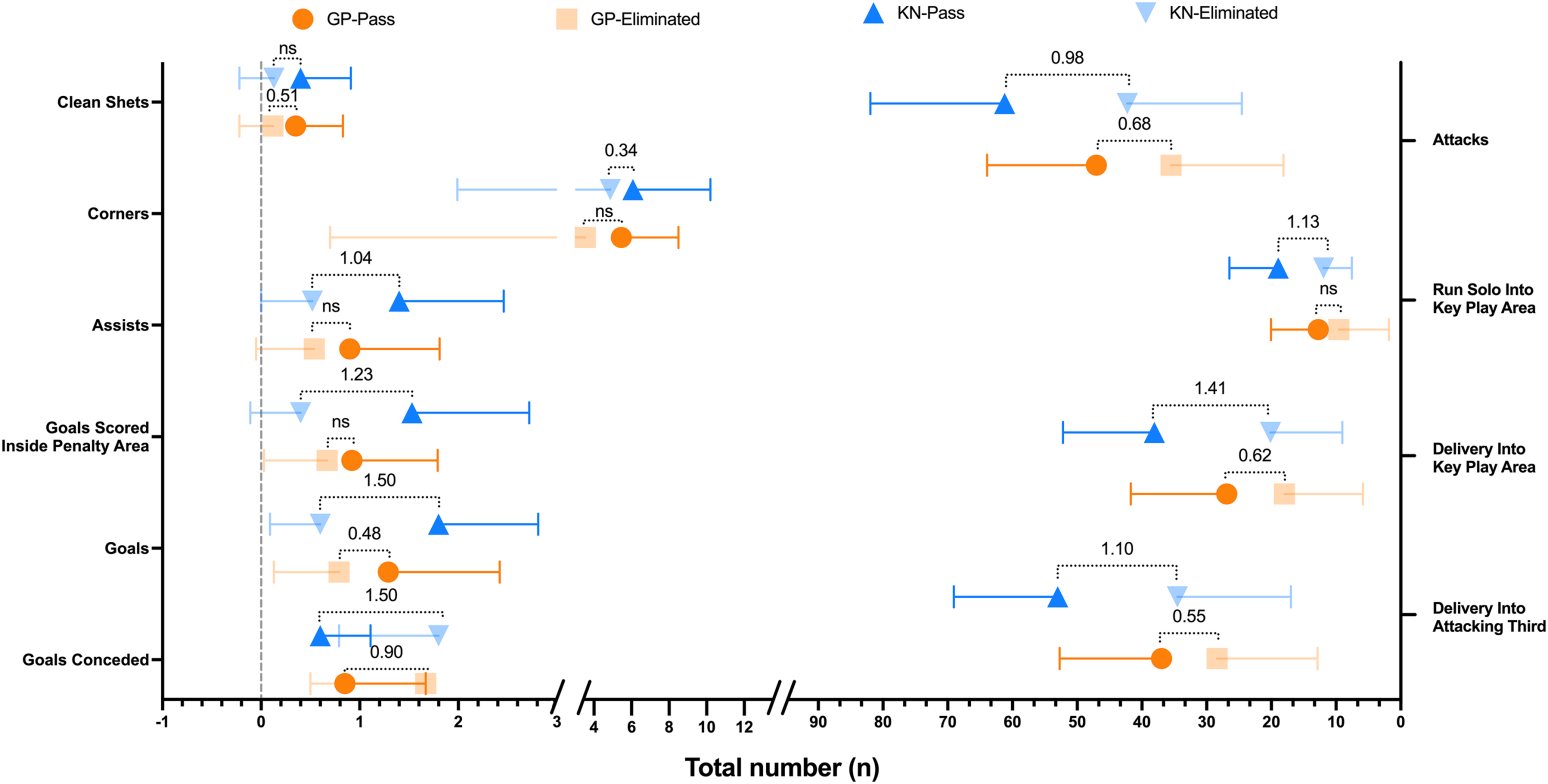
Goalkeeping, attacking and distribution related significant variables included in the generalized linear model. Values inside de graph represent the effect size (*η*^2^p); GP, group phase; KN, knockout phase; ns, non-significant.

**Figure 3:**
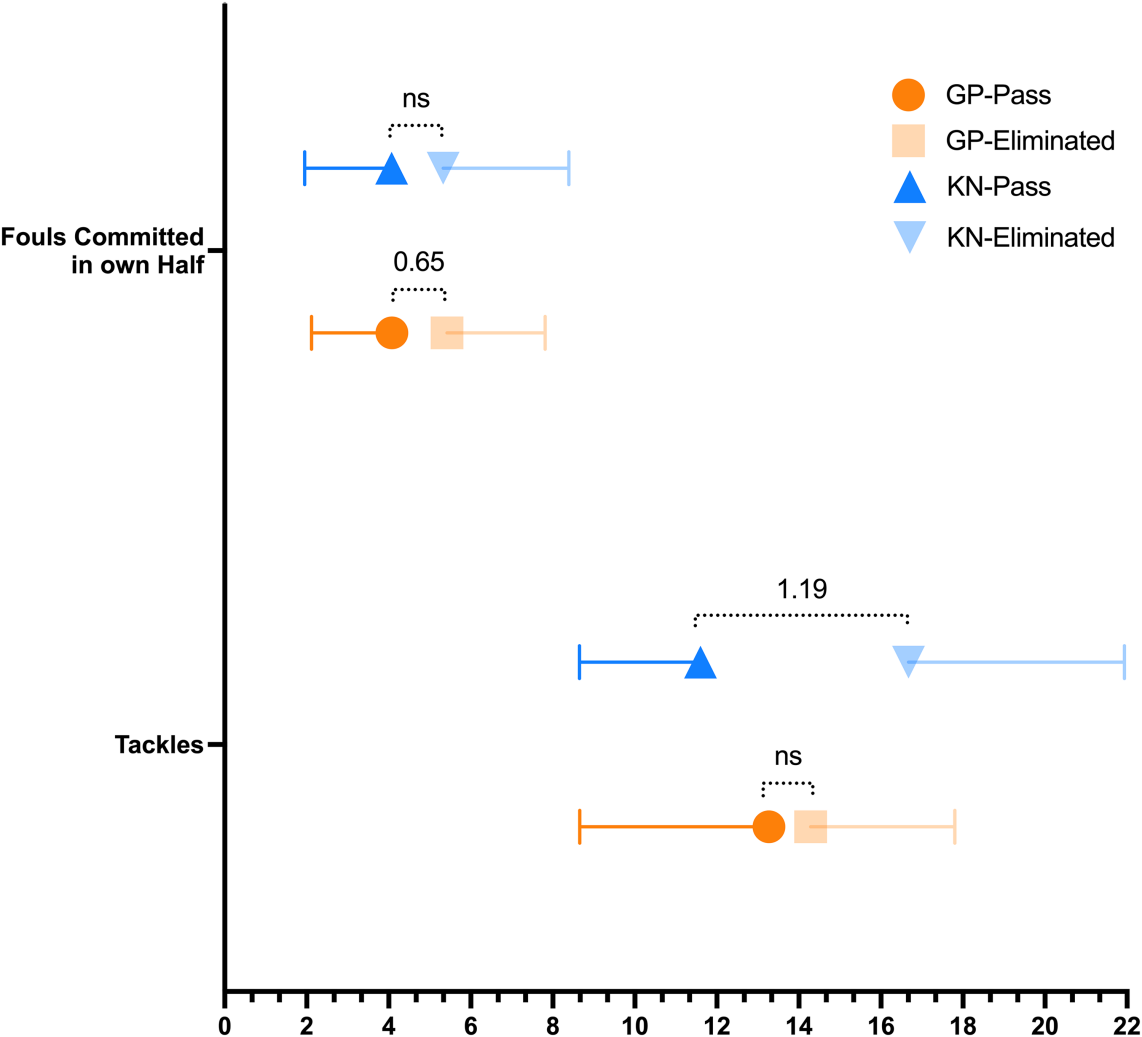
Defensive and disciplinary related significant variables included in the generalized linear model. Values inside de graph represent the effect size (*η*^2^p); GP, group phase; KN, knockout phase; ns, non-significant.

Regarding the group stage, the variables related to distribution revealed that teams advancing to the knockout phase exhibited significantly higher values in Ball Possession (%) (*η²p* = 0.086; *d* = 0.64), Delivery Into Attacking Third (*η²p* = 0.064; *d* = 0.55), Delivery Into Key Play Area (*η²p* = 0.085; *d* = 0.62), Passes Attempted (*η²p* = 0.089; *d* = 0.64), Passes Completed (*η²p* = 0.087; *d* = 0.63), Passes Short Completed (*η²p* = 0.103; *d* = 0.69), Passes Medium Completed (*η²p* = 0.060; *d* = 0.52), Passes Completed Backward (*η²p* = 0.145; *d* = 0.83), Passes Completed To Left (*η²p* = 0.058; *d* = 0.51), and Passes Completed To Right (*η²p* = 0.056; *d* = 0.50) (Figures 1, 2). In relation to attacking variables, significant differences were found for Attacks (*η²p* = 0.095; *d* = 0.68) (Figure 2), Corners (*η²p* = 0.086; *d* = 0.64), and Goals (*η²p* = 0.054; *d* = 0.48). Regarding goalkeeping, significant differences were found for Goals Conceded (*η²p* = 0.145; *d* = 0.90) and Clean Sheets (*η²p* = 0.058; *d* = 0.51) (Figure 3). Finally, for disciplinary variables, differences were significant only for Fouls Committed in Own Half (*η²p* = 0.058; *d* = 0.65) (Figure 3). On the Other hand, no effect was found for defensive variables. We can check all the variables in Table 1.

**Table 1:** Analysis of the results based on whether the teams were eliminated or advanced in the tournament stage.

	Groups phase				Knockout phase			
	Eliminated	Not eliminated	F	*p* value	Eliminated	Not eliminated	F	*p* value
Distribution								
Ball possession (%)	**45.83** ± **9.49**	**52.08** ± **9.84**	**6**.**608**	**0**.**012**	**44.07** ± **9.24**	**55.93** ± **9.24**	**12**.**4**	**0**.**002**
Free kick	12.75 ± 3.69	12.35 ± 3.56	0.193	0.661	12.00 ± 3.82	13.07 ± 5.96	0.341	0.564
Delivery into attacking third	**28.38** ± **15.52**	**36.96** ± **15.76**	**4**.**794**	**0**.**032**	**34.53** ± **17.55**	**53.00** ± **16.05**	**9**.**045**	**0**.**006**
Delivery into key play area	**18.00** ± **12.14**	**26.90** ± **14.82**	**6**.**465**	**0**.**013**	**20.13** ± **11.09**	**38.07** ± **14.13**	**14**.**953**	**0**.**001**
Cross attempted	16.83 ± 9.04	16.52 ± 6.98	0.026	0.872	17.67 ± 10.02	19.13 ± 9.20	0.174	0.679
Cross accuracy (%)	22.62 ± 11.66	26.67 ± 12.80	1.689	0.198	23.20 ± 11.75	22.33 ± 10.83	0.044	0.835
Cross completed	3.83 ± 2.56	4.38 ± 2.29	0.824	0.367	4.53 ± 3.85	4.20 ± 2.73	0.075	0.787
Passes attempted	**418.50** ± **112.45**	**503.81** ± **138.40**	**6**.**844**	**0**.**011**	**464.27** ± **164.05**	**602.07** ± **125.44**	**6**.**679**	**0**.**015**
Passes completed	**355.21** ± **112.75**	**440.85** ± **141.27**	**6**.**677**	**0**.**012**	**398.80** ± **167.95**	**542.13** ± **119.25**	**7**.**263**	**0**.**012**
Passes accuracy (%)	83.70 ± 5.59	86.27 ± 5.25	3.647	0.06	**84.27** ± **7.42**	**89.80** ± **3.14**	**7**.**071**	**0**.**013**
Passes short completed	**87.71** ± **31.57**	**118.65** ± **48.46**	**8**.**042**	**0**.**006**	**105.73** ± **44.64**	**134.20** ± **29.44**	**4**.**25**	**0**.**049**
Passes medium completed	**237.21** ± **86.45**	**287.15** ± **98.64**	**4**.**439**	**0**.**039**	**262.27** ± **122.61**	**367.13** ± **97.97**	**6**.**7**	**0**.**015**
Passes completed backward	**64.54** ± **18.25**	**86.94** ± **28.99**	**11**.**908**	**0**.**001**	**68.53** ± **30.93**	**103.93** ± **21.03**	**13**.**433**	**0**.**001**
Passes completed to left	**99.29** ± **36.46**	**120.19** ± **41.85**	**4**.**332**	**0**.**041**	**112.93** ± **53.70**	**149.67** ± **40.35**	**4**.**486**	**0**.**043**
Passes completed to right	**99.25** ± **38.74**	**120.73** ± **43.77**	**4**.**149**	**0**.**045**	112.27 ± 51.80	147.20 ± 43.94	3.97	0.056
Attacking								
Attacks	**35.50** ± **17.37**	**47.04** ± **16.87**	**7**.**347**	**0**.**008**	**42.27** ± **17.72**	**61.20** ± **20.75**	**7**.**22**	**0**.**012**
Corners	**3.54** ± **2.84**	**5.46** ± **3.04**	**6**.**616**	**0**.**012**	4.87 ± 2.88	6.07 ± 4.13	0.853	0.364
Goals	**0.79** ± **0.66**	**1.29** ± **1.13**	**4**.**007**	**0**.**049**	**0.60** ± **0.51**	**1.80** ± **1.01**	**16**.**8**	**0**.**001**
Goals scored inside penalty area	0.67 ± 0.64	0.92 ± 0.87	1.556	0.216	**0.40** ± **0.51**	**1.53** ± **1.19**	**11**.**6**	**0**.**002**
Attempts off target	3.92 ± 2.26	4.90 ± 2.64	2.406	0.125	4.93 ± 2.81	6.60 ± 4.34	2.406	0.125
Attempts off target outside penalty area	1.50 ± 1.18	1.90 ± 1.21	1.746	0.191	1.60 ± 1.30	3.07 ± 2.02	1.746	0.191
Attempts on target outside penalty area	1.46 ± 1.14	1.54 ± 1.37	0.066	0.798	0.80 ± 0.68	1.73 ± 1.44	0.066	0.798
Assists	0.54 ± 0.59	0.90 ± 0.91	3.025	0.086	**0.53** ± **0.52**	**1.40** ± **1.06**	**8**.**16**	**0**.**008**
Run solo into key play area	9.58 ± 7.76	12.75 ± 7.30	2.888	0.094	**11.93** ± **4.37**	**18.93** ± **7.56**	**9**.**636**	**0**.**004**
Defending								
Tackles	14.29 ± 3.51	13.27 ± 4.61	0.912	0.343	**16.67** ± **5.27**	**11.60** ± **2.95**	**10**.**551**	**0**.**003**
Tackles won	5.25 ± 2.69	5.17 ± 3.17	0.012	0.912	6.80 ± 3.90	5.27 ± 2.28	1.73	0.199
Clearance attempted	20.96 ± 7.83	18.38 ± 8.52	1.55	0.217	20.73 ± 9.25	21.20 ± 14.45	0.011	0.917
Clearance completed	17.17 ± 6.73	14.29 ± 7.39	2.568	0.114	15.80 ± 7.47	16.20 ± 10.46	0.015	0.905
Goalkeeping								
Goals conceded	**1.67** ± **1.17**	**0.85** ± **0.82**	**11**.**7**	**0**.**001**	**1.80** ± **1.01**	**0.60** ± **0.51**	**16**.**8**	**0**.**001**
Clean sheet	**0.12** ± **0.34**	**0.35** ± **0.48**	**4**.**324**	**0**.**041**	0.13 ± 0.35	0.40 ± 0.51	2.8	0.105
Claims	2.46 ± 1.77	2.29 ± 1.61	0.160	0.690	3.73 ± 1.49	2.93 ± 2.37	1.22	0.278
Claims high	1.17 ± 1.20	1.15 ± 1.07	0.006	0.941	1.73 ± 1.28	1.27 ± 1.22	1.04	0.316
Claims low	1.29 ± 1.20	1.12 ± 1.12	0.337	0.563	1.93 ± 1.33	1.67 ± 1.68	0.232	0.634
Punches	0.75 ± 0.74	0.52 ± 0.88	1.243	0.274	0.87 ± 1.55	0.53 ± 0.74	0.563	0.459
Disciplinary								
Fouls committed	11.71 ± 3.94	10.98 ± 3.12	0.732	3.95	12.07 ± 5.05	9.80 ± 3.36	2.094	0.159
Fouls committed in defensive third	2.12 ± 1.26	1.73 ± 1.25	1.594	0.211	2.87 ± 2.29	2.13 ± 1.55	1.05	0.314
Fouls committed in own half	**5.42** ± **2.39**	**4.08** ± **1.96**	**6**.**392**	**0**.**014**	5.33 ± 3.06	4.07 ± 2.12	1.73	0.199

Those in bold indicate significant differences.

During the knockout stage, several additional variables became significant across different categories. For distribution, significant variables included Ball Possession (%) (*η²p* = 0.306; *d* = 1.28), Delivery Into Attacking Third (*η²p* = 0.244; *d* = 1.10), Delivery Into Key Play Area (*η²p* = 0.348; *d* = 1.41), Passes Attempted (*η²p* = 0.193; *d* = 0.94), Passes Completed (*η²p* = 0.206; *d* = 0.98), Passes Accuracy (*η²p* = 0.202; *d* = 0.97), Passes Short Completed (*η²p* = 0.132; *d* = 0.75), Passes Medium Completed (*η²p* = 0.193; *d* = 0.94), Passes Completed Backward (*η²p* = 0.324; *d* = 1.34), and Passes Completed to Left (*η²p* = 0.138; *d* = 0.77) (Figure 1).

Conversely, Passes Completed to the Right lost significance. Regarding Attacking, in this category, significant variables included Attacks (*η²p* = 0.205; *d* = 0.98) (Figure 1), Goals (*η²p* = 0.375; *d* = 1.50), Goals Scored Inside the Penalty Area (*η²p* = 0.292; *d* = 1.23), Assists (*η²p* = 0.226; *d* = 1.04), Run solo Into Key Play Area (*η²p* = 0.256; *d* = 1.13), on the other hand, the variable Corners is no longer significant. Some defensive variables become significant too, specifically, Tackles (*η²p* = 0.274; *d* = 1.19) (Figure 3). Regarding Goalkeeping only Goals Conceded had significant effect (*η²p* = 0.375; *d* = 1.50), meanwhile Clean Sheets lost significance in this stage (Figure 2). Lastly, regarding disciplinary no significant changes or new significant variables were reported in this category during the knockout stage. Once again, we can check all the variables in Table 1. It is important to note that at this stage, most effect sizes were considered large, in contrast to the earlier stage, where only Goals Conceded and Backward Passes Completed had large effect sizes.

## 4 Discussion

The study aimed to identify the KPI that distinguish football teams advancing from the group stage from those that do not, as well as those progressing through the knockout stage from those that fail to advance during the EURO 2024. The main findings were: (i) during the group stage, teams advancing to the knockout rounds demonstrated superior performance across distribution, attacking, goalkeeping and disciplinary variables, but not for defending elements, pointing out the Goals Conceded and Passes Completed Backward due to their large effect size on overall performance (ii) in the knockout rounds, although the distribution and goalkeeping categories remain important, the number of variables related to attacking and defending increases to distinguish teams that progress from those that do not, while disciplinary becomes less significant.

### 4.1 Group stage

During the group stage, teams advancing to the knockout rounds consistently demonstrated superior performance across a range of variables. Notably, Goals Conceded emerged as a key variable with large effect size, emphasizing the importance of conceding a low number of goals to progress in the competition, a factor reinforced by the significance of clean sheets in this phase. These variables, categorized by UEFA under Goalkeeping, were identified as individual performance factors for goalkeepers in previous literature (27), and could exhibit a direct transfer, due to the inherent impact of goals on matches and rankings (17, 21), as they also contribute to scenarios involving point ties between teams.

In addition, we can also highlight the importance of efficient game distribution. Although Passes Completed Backward presented a large effect size, other variables such as Ball Possession (%), Passes (Attempted, Completed, Short Completed, Medium Completed, Completed To Left and Right) and Deliveries (Into Attacking Third and Key Play Area). These metrics highlight the critical role of ensuring controlled build-up play to retain possession and progress in offensive play, this game model, where these variables play a prominent role, is frequently employed in the analysis of national competitions (9, 28), and in some cases, it can be associated with teams that show better performance in tournaments (29). However, possession is one of the most debated variables in performance analysis, as its influence on performance yields mixed results (8, 30), this comparison highlights it as an important factor for differentiating between these teams, aligning with studies such as Jerome et al. (30), which highlight its significant impact on football performance.

In this sense, the role of Passes Completed Backward also stands out, as they appear to play a significant role in maintaining tactical structure and controlling the game in highly competitive contexts such as the EURO. Although offensive progression and forward passing are often more highly valued (31), these findings suggest that effective use of backward passes may be associated with more successful teams. Strategically, backward passes contribute to retaining possession, resetting play in an organized manner, and managing the game's tempo. Furthermore, they help preserve team structure, reduce the risk of turnovers in dangerous areas, and support more elaborate offensive build-up. This allows teams to gain more time to identify and exploit defensive gaps, particularly when facing defensive low-blocks (9).

Additionally, the number of attacks and goals also showed a clear relationship with team progression, with higher values observed in teams that advanced to the next round. This finding is widely supported by the existing literature (13, 20, 21, 29).

Lastly, both a higher number of corners and fewer fouls committed in their own half can be considered relevant factors, as they may reflect the overall trend of the match. Although both variables may be related to set piece situations (such as corners and direct free kicks), which clearly offer goal-scoring opportunities for the executing team (32, 33), corners in particular have been highlighted as highly relevant in previous research (21), specifically on this type of tournament (34), primarily focused on how goals are scored (18, 35).

In relation to previous research, our findings align with those of Dufour et al. (21), who reported that, in the context of a World Cup, teams advancing from the group stage tended to score more goals. While we did not observe statistically significant differences in most of the remaining offensive variables, our analysis suggests that a higher volume of attacking actions may be associated with an increased likelihood of progressing beyond the group stage. Additionally, despite using a dataset similar to that employed by Stafylidis et al. (22), the key performance indicators identified differed considerably, with deliveries into the attacking third being the only variable common to both analyses. This discrepancy may be attributed to the fact that their primary comparison focused on match outcomes and the regression model applied, rather than on progression through the group stage.

### 4.2 Knockout stage

In the knockout rounds, the relevance of certain variables shifted, reflecting the heightened tactical and strategic demands of elimination games. Variables such as Clean Sheet, Corners, Fouls Committed in own Half and Passes Completed To Right lost their significance, suggesting that the impact of these variables could be comparable among the teams that advance to the knockout stage.

Although many game distribution variables appear consistent across teams, their relevance remains high and their effect sizes even increase, with Pass Accuracy standing out as an important factor in distinguishing team progression in the knockout stage. Variables such as ball possession and pass accuracy have been associated with success in football in previous research (8, 36), being decisive even in shaping playing styles across different European leagues (9, 37, 38), although it does not seem to have been as decisive in success during the previous World Cups (39), where other playing styles, focused on direct play and pressing, being predominant. This metric has also received considerable attention in studies focused on goal scoring across various leagues, further reinforcing its relevance in offensive performance (40). The playing styles of countries from other leagues or continents, which have not yet been thoroughly examined and defined, may play a significant role in this determination. Additionally, it is also noteworthy that completed passes to the right lose relevance at this stage, a trend that appears to align with previous studies highlighting a greater prevalence of attacks through the left and central zones (41).

However, in the attacking category, we observe a significant shift with the increasing relevance of differences in Assists, Solo Runs Into Key Play Area, and Goals Scored Inside Penalty Area. In studies such as Yan et al. (42) on the 2022 World Cup, various winning scenarios are examined, with one of the predominant models being based on ball possession, successful breakthroughs against the opponent's defensive line, and receptions in the final third. These metrics closely align with those identified in the present study and are further supported by others such as Deb et al. (31) or Kyranoudis et al. (43) where it can be observed that both passing and actions near the penalty area take on particular importance in the context of goal scoring, which remains one of the key factors alongside the number of attacks, as also observed during the group stage.

This pattern is particularly evident during the knockout phase, where teams advancing through multiple rounds tend to reinforce and even bias the model, as the more matches a team wins, the greater its presence and influence on the overall performance trends. Spain and England serve as illustrative examples. The persistence of these metrics across tournament stages, alongside the continued importance of distribution-related variables, suggests that a key distinction between advancing and non-advancing teams may lie in their ability to sustain effective possession phases and construct offensive play. This, in turn, reinforces the importance of maintaining both offensive effectiveness and tactical consistency during the critical stages of the competition.

Similarly, the defensive phase exhibits significant differences at this stage, which were not observed during the group phase. Tackles appear to be the variable that differentiates between elimination and progression. This may suggest that the more successful teams in this phase tend to maintain greater possession and engage in fewer defensive interventions. These findings align with previous research (44, 45), where these differences are explained by more defence-oriented mentalities or defensive playing styles (46), which often contrast with ball possession, a factor that appears to distinguish the teams that advance in these knockout rounds.

Finally, we observe that variables related to disciplinary behaviour decrease in importance during this phase, as do those related to goalkeeping. However, goals conceded, given their inherent significance, have remained relevant in this stage.

## 5 Limitations and future research

Several limitations should be acknowledged when interpreting the findings of this study. First, although all matches from the final phase of UEFA EURO 2024 were included, the analysis was conducted at the match level, meaning that differences in the number of matches played by each team were not accounted for beyond treating each performance independently. Second, matches that included red cards were not excluded, as the aim was to maintain ecological validity and reflect the full competitive reality of the tournament; however, dismissals may have influenced certain performance indicators and should be considered when interpreting the results. Third, although the performance data were obtained from official and publicly available UEFA sources, working with secondary data implies limited influence over the way certain variables were originally defined or reported.

Building on the limitations acknowledged in this study, several avenues for future research can be proposed. First, it would be valuable to deepen the understanding of playing patterns and attempt to develop structured models of play. Although we acknowledge the limitations inherent to the sample size in this type of tournament context, such an approach could provide insights that are currently lacking in the literature. This line of inquiry, especially when combined with longitudinal tracking of team or player performance across multiple competitions, could help explore the consistency and evolution of playing styles and tactical approaches over time. Second, incorporating contextual variables such as opposition strength, match status (e.g., leading or trailing), or game location (home/away/neutral) could enhance the interpretation of performance indicators and their impact on match outcomes. Third, future studies may benefit from integrating tracking data (e.g., player positioning, movement patterns) to complement event-based performance metrics and provide a more nuanced understanding of tactical behaviors.

Additionally, qualitative approaches involving expert analysis from coaches or analysts could enrich the interpretation of quantitative data.

## 6 Practical applications

For coaches and analysts, these results offer valuable insights for optimizing team performance at different stages of competitive play, fostering adaptability involves preparing teams for a range of scenarios. In the group stage, the relevance of accurate distribution highlights the importance of reinforcing controlled build-up during games. In this regard, previous studies have emphasized the relevance of possession or build-up based playing styles as a successful approach in similar contexts (9). In contrast, the knockout stage demands greater pass accuracy, attacking efficiency, and defensive effectiveness, suggesting the need to design training scenarios that replicate high-pressure environments and decision-making under stress. In such contexts, teams are required to be extremely precise and to minimize errors in both offensive and defensive phases, as this precision may be what ultimately separates champion teams from those that are eliminated. Specific attention should be given to actions in the final third, such as assists, solo runs, and shots from inside the penalty area, as these variables were more strongly associated with successful outcomes in the later stages.

However, it is essential to recognize that performance is influenced by a multitude of factors and can fluctuate throughout the tournament (46, 47). Consequently, we cannot ascertain a singular “path to success”. In that way, building a team with players who can perform effectively under varying tactical demands and match scenarios is critical for tournament success, considering not only for training but also in the squad selection and roster construction.

## 7 Conclusion

This study provides new insights into the performance indicators that distinguish successful teams in the UEFA EURO 2024, considering both the group and knockout stages as separate competitive contexts. During the group stage, variables such as Goals Conceded and Passes Completed Backward had the most substantial influence, highlighting the importance of keeping conceded goals to a minimum and controlling ball distribution at this stage. Other factors, such as goals scored, number of attacks, corners, and fouls committed, also showed significant differences between qualified and eliminated teams, although with comparatively smaller effects.

In contrast, knockout stage matches were characterized by greater precision in offensive play, with particular emphasis on actions in the final third. Tackles also emerged as a relevant variable. Variables related to ball distribution remained important, while those associated with disciplinary aspects and goalkeeping decreased in influence. However, goals scored and goals conceded persisted as consistent performance indicators across both stages of the tournament.

## Data Availability

The original contributions presented in the study are included in the article; additional queries can be addressed to the corresponding author. All data collected is available at: https://www.uefa.com/euro2024/fixtures-results/#/d/2024-07-14.

## References

[1] Romero-Rodríguez R-C Alonso-Pérez-Chao E Ribas C Memmert D Gómez-Ruano M-Á. Influence of contextual factors on most demanding scenarios in under-19 professional soccer players. Biol Sport. (2024) 41(4):51–60. 10.5114/biolsport.2024.136087 39416511 PMC11475010

[2] Lago C Casais L Dominguez E Sampaio J. The effects of situational variables on distance covered at various speeds in elite soccer. Eur J Sport Sci. (2010) 10(2):103–9. 10.1080/17461390903273994

[3] Castellano J. Relación entre indicadores de rendimiento y el éxtio en el fútbol profesional. Rev Iberoam Psicol del Ejerc y el Deport. (2018) 13(1):41–9. https://www.redalyc.org/articulo.oa?id=311153534004

[4] García-Rubio J García-Vallejo A Arenas-Pareja M de los Á López-Sierra P Ibáñez SJ. From junior to elite in soccer: exploring the relative age effect and talent selection in Spanish youth national teams. Children. (2022) 9(10):1543. 10.3390/children9101543 36291479 PMC9600190

[5] Clemente FM. Study of successful soccer teams on FIFA world cup 2010. Pamukkale J Sport Sci. (2012) 3(3):90–103. https://dergipark.org.tr/en/pub/psbd/issue/20578/219258#article_cite

[6] Del Coso J Brito de Souza D López-Del Campo R Blanco-Pita H Resta R. The football championship is won when playing away: difference in match statistics between the winner and the second-place team in LaLiga. Int J Perform Anal Sport. (2020) 20(5):879–91. 10.1080/24748668.2020.1801201

[7] Coppola R Pignato S D’ambrosio G Lipoma M. Analysis of discriminant performance indicators of the FIFA ranking in the 2018 world cup. J Phys Educ Sport. (2019) 19(5):1820–4. 10.7752/jpes.2019.s5267

[8] Lepschy H Wäsche H Woll A. Success factors in football: an analysis of the German bundesliga. Int J Perform Anal Sport. (2020) 20(2):150–64. 10.1080/24748668.2020.1726157

[9] Martín-Castellanos A Flores MR Solana DM Del Campo RL Garrosa FN Mon-López D. How do the football teams play in LaLiga? Analysis and comparison of playing styles according to the outcome. Int J Perform Anal Sport. (2024) 24(1):18–30. 10.1080/24748668.2023.2262813

[10] Branquinho L França E Teixeira J Valente N Reis T Thomatieli-Santos RV Comparing physical, technical and tactical performances in the world cup Qatar 2022. J Hum Sport Exerc. (2024) 19(2):654–66. 10.55860/t8ybtk14

[11] Winter C Pfeiffer M. Tactical metrics that discriminate winning, drawing and losing teams in UEFA EURO 2012®. J Sports Sci. (2016) 34(6):486–92. 10.1080/02640414.2015.1099714 26508419

[12] Braga JAL Moreira PG de Sousa PG da Costa VT. Identification of indicators that predict victory in the five main CONMEBOL and UEFA leagues. Int J Sports Sci Coach. (2024) 19(5):2078–89. 10.1177/17479541241249475

[13] Sgro F Barresi M Lipoma M. The analysis of discriminant factors related to team match performances in European football championship 2012. J Phys Educ Sport. (2015) 15(3):460–5. 10.7752/jpes.2015.03069

[14] De Amorim Mendes MH Rezende VHS Praça GM. Association between goal kick strategies and the offensive outcome in the UEFA EURO 2020. Hum Mov. (2023) 24(3):64–70. 10.5114/hm.2023.125924

[15] Amatria M Maneiro-Dios R Anguera MT. Analysis of the success of the Spanish national football team in the UEFA EURO 2012. Apunt Educ Fis y Deport. (2019) 3(137):85–102. 10.5672/apunts.2014-0983.es.(2019/3).137.07

[16] Muhamad S Norasrudin S Rahmat A. Differences in goal scoring and passing sequences between winning and losing team in UEFA-EURO championship 2012. World acad sci eng technol int sci index 74. Int J Soc Educ Econ Manag Eng. (2013) 7(2):224–9. 10.1177/0193723512467192

[17] Brito de Souza D López-Del Campo R Blanco-Pita H Resta R Del Coso J. An extensive comparative analysis of successful and unsuccessful football teams in LaLiga. Front Psychol. (2019) 10:1–8. 10.3389/fpsyg.2019.02566 31781011 PMC6856952

[18] Muriarte Solana D Gallardo Marmol F Grande Rodriguez I Barba-Ruiz M Hernández-Lougedo J Martín-Castellanos A. Comparativa de los goles marcados a balón parado durante la eurocopa y la Copa América 2021. Apunt Educ Física y Deport. (2023) 4(154):95–107. 10.5672/apunts.2014-0983.es.(2023/4).154.09

[19] Kubayi A Toriola A. Differentiating African teams from European teams: identifying the key performance indicators in the FIFA world cup 2018. J Hum Kinet. (2020) 73(1):203–8. 10.2478/hukin-2019-0144 32774551 PMC7386154

[20] Karataş A Inan T. The team ranking and match performance analysis in WORLD CUP. Dokuz Eylül Üniversitesi Spor Bilim Derg. (2024) 2(1):66–79. 10.5281/zenodo.12592191

[21] Dufour M Phillips J Ernwein V. What makes the difference? Analysis of the 2014 world cup. J Hum Sport Exerc. (2017) 12(3):616–29. 10.14198/jhse.2017.123.06

[22] Stafylidis A Mandroukas A Michailidis Y Metaxas TI. Decoding success: predictive analysis of UEFA EURO 2024 to uncover key factors influencing soccer match outcomes. Appl Sci. (2024) 14(17):7740. 10.3390/app14177740

[23] Union of European Football Associations. Fixtures & results | UEFA EURO 2024 | UEFA.com. (2024) Available online at: https://www.uefa.com/euro2024/fixtures-results/#/d/2024-07-14 (Accessed December 17, 2024).

[24] Kong L Zhang T Zhou C Gómez MÁ Hu Y. The evaluation of playing styles integrating with contextual variables in professional soccer. Front Psychol. (2022) 13:1–10. 10.3389/fpsyg.2022.1002566 PMC9539538 36211871

[25] Pierce CA Block RA Aguinis H. Cautionary note on reporting eta-squared values from multifactor anova designs. Educ Psychol Meas. (2004) 64(6):916–24. 10.1177/0013164404264848

[26] Cohen J. Statistical Power Analysis of the Behavioral Sciences. 2nd edn New York: Academic Press (1988).

[27] Tienza-Valverde A Hernández-Beltrán V Espada MC Bravo-Sánchez A Santos FJ Gamonales JM. Analysis of individual performance indicators of football goalkeeper. Apunt Sport Med. (2023) 58(219):1–12. 10.1016/j.apunsm.2023.100420

[28] Fernández Navarro J. Analysis of styles of play in soccer and their effectiveness* (Doctoral thesis)*. Granada: Universidad de Granada (2018). https://digibug.ugr.es/bitstream/handle/10481/54554/61550.pdf

[29] Stafylidis A Mandroukas A Papadopoulou SD Michailidis Y Kyranoudis A Gissis Ι Analysis of game-related performance indicators in the Greek soccer league: insights from the 2020-2021 season. Trends Sport Sci. (2024) 31(1):37–44. 10.23829/TSS.2024.31.1-4

[30] Jerome BWC Stoeckl M Mackriell B Dawson CW Fong DTP Folland JP. Evidence for a new model of the complex interrelationship of ball possession, physical intensity and performance in elite soccer. Scand J Med Sci Sport. (2024) 34(1):1–13. 10.1111/sms.14546 38059701

[31] Deb B Fernandez-Navarro J Jarman I Mcrobert AP. Creating an augmented possession framework to evaluate phases of play and application in international football. J Sport Anal. (2024) 10(0):1–11. 10.1177/22150218241290988

[32] Sarmento H Afonso J Clemente F Gouveia ÉR Ordoñez-Saavedra N Silva J Unlocking the power of set pieces in men’s professional football – a scoping review. Int J Sports Med. (2025) 46(8):537–61. 10.1055/a-2563-0323 40216395

[33] Li C Zhao Y. Comparison of goal scoring patterns in “the big five”. European Football Leagues. Front Psychol. (2021) 11:1–7. 10.3389/fpsyg.2020.619304 PMC7838214 33519643

[34] Kubayi A Larkin P. Analysis of teams’ corner kicks defensive strategies at the FIFA world cup. Int J Perform Anal Sport. (2019) 19(5):809–19. 10.1080/24748668.2019.1660547

[35] Ardá T Maneiro R Rial A Losada JL Casal CA. Análisis de la eficacia de los saques de esquina en la copa del mundo de fútbol 2010. Un intento de identificación de variables explicativas. Rev Psicol del Deport. (2014) 23(1):165–72. https://ruc.udc.es/entities/publication/b9246d6d-cd77-4bcd-8b41-f3e64b130e66

[36] González-Rodenas J Ferrandis J Moreno-Pérez V López-Del Campo R Resta R Del Coso J. Differences in playing style and technical performance according to the team ranking in the Spanish football LaLiga. A thirteen seasons study. PLoS One. (2023) 18:1–15. 10.1371/journal.pone.0293095 PMC10588845 37862370

[37] Fernandez-Navarro J Fradua L Zubillaga A Ford PR McRobert AP. Attacking and defensive styles of play in soccer: analysis of Spanish and English elite teams. J Sports Sci. (2016) 34(24):2195–204. 10.1080/02640414.2016.1169309 27052355

[38] Plakias S Moustakidis S Kokkotis C Tsatalas T Papalexi M Plakias D Identifying soccer Teams’ styles of play: a scoping and critical review. J Funct Morphol Kinesiol. (2023) 8(2):39. 10.3390/jfmk8020039 37092371 PMC10123610

[39] Lepschy H Woll A Wäsche H. Success factors in the FIFA 2018 world cup in Russia and FIFA 2014 world cup in Brazil. Front Psychol. (2021) 12:1–9. 10.3389/fpsyg.2021.638690 PMC7985168 33767649

[40] Prieto-González P Martín V Pacholek M Sal-de-Rellán A Marcelino R. Impact of offensive team variables on goal scoring in the first division of the Spanish soccer league: a comprehensive 10-year study. Sci Rep. (2024) 14(1):25231. 10.1038/s41598-024-77199-8 39448790 PMC11502709

[41] Kempe M Vogelbein M Nopp S. The cream of the crop: analysing FIFA world cup 2014 and Germany’s title run. J Hum Sport Exerc. (2016) 11(1):42–52. 10.14198/jhse.2016.111.04

[42] Yan W Li S Wang D Yuan B Zeng H Ren D. How to win in FIFA world cup Qatar 2022? A study on the configurations of technical and tactical indicators based on fuzzy-set qualitative comparative analysis. Front Psychol. (2023) 14:1–10. 10.3389/fpsyg.2023.1307346 PMC10853467 38343895

[43] Kyranoudis E Papadimitriou K Kyranoudis A Giannakopoulos A Kontstantinidou X. Final pass and its relationship with final action for the creation of goal-scoring opportunities at EURO 2020. J Hum Sport Exerc. (2024) 19(2):499–509. 10.55860/hf7g0141

[44] Lepschy H Wäsche H Woll A. How to be successful in football: a systematic review. Open Sports Sci J. (2018) 11(1):3–23. 10.2174/1875399X01811010003

[45] Renner V Görgen K Woll A Wäsche H Schienle M. Success factors in national team football: an analysis of the UEFA EURO 2020. J Quant Anal Sport. (2024) 21(1):71–93. 10.1515/jqas-2023-0026

[46] Bialkowski A Lucey P Carr P Yue Y Sridharan S Matthews I. Identifying team style in soccer using formations learned from spatiotemporal tracking data. IEEE Int Conf Data Min Work. (2014):9–14. 10.1109/ICDMW.2014.167

[47] Yi Q Gómez MA Wang L Huang G Zhang H Liu H. Technical and physical match performance of teams in the 2018 FIFA world cup: effects of two different playing styles. J Sports Sci. (2019) 37(22):2569–77. 10.1080/02640414.2019.1648120 31354060

